# Arabic Aphasia Research Through a Clinical and Linguistic Lens: A Systematic Review of Current Limitations and Future Directions

**DOI:** 10.1111/1460-6984.70064

**Published:** 2025-06-16

**Authors:** Tariq Khwaileh, Eiman Mustafawi, Shereen Elbuy, Noor Numan, Samawiyah Ulde

**Affiliations:** ^1^ Department of English Literature and Linguistics Qatar University Doha Qatar; ^2^ Department of Linguistics University of Potsdam Potsdam Germany; ^3^ Department of Patient Experience Sidra Medicine Doha Qatar

**Keywords:** aphasia, cross‐linguistic, brain damage, morphology, assessment, speech and language therapy

## Abstract

**Background:**

Aphasia has been widely investigated for English and other Indo‐European languages such as German, Dutch, Italian and Spanish. It has been reported that published studies on Arabic aphasia only comprised five studies, accounting for only 0.40% of the total literature on aphasia between 2000 and 2009.

**Aims:**

The present paper is a systematic review of studies that have been published on Arabic aphasia. The main objective of this study is to review the body of aphasia literature on Arabic, to identify strengths and weaknesses in the available clinical resources for Arabic aphasia.

**Methods:**

Preferred Reporting Items for Systematic Reviews and Meta‐Analyses (PRISMA) guidelines were followed. Five relevant databases were identified and searched using predefined keywords. A 6th source, Google Scholar, was also used to yield grey literature; these sources were screened on Scimago for quality assurance. Predefined eligibility criteria were then applied to the records that the initial search yielded. The final list of included studies was then qualitatively reviewed.

**Main Contribution:**

The search yielded 48 studies. The resulting review identified a scarcity of research on assessment materials, efficacy of therapy and interventions and linguistic/psycholinguistic theory that underscores the development of clinical resources for Arabic aphasia. It suggested specific areas where development is required in each category. From the available research, it identified limitations in available materials for clinical assessment in Arabic aphasia. Specifically, that currently available materials are (a) primarily translations and adaptations of other languages rather than being developed with linguistically and culturally specific features of Arabic in mind; (b) are screening and short assessments rather than comprehensive batteries and (c) are not controlled for crucial psycholinguistic variables such as imageability and age of acquisition.

**Conclusions:**

While research on Arabic aphasia has been growing in the past few years, it lacks in several areas of investigation, including certain methodological approaches, the varieties investigated, aphasia types and the formulation of valid assessment protocols and therapy interventions.

**WHAT THIS STUDY ADDS:**

*What is already known on this subject*

- Aphasia has been widely investigated for English and other Indo‐European languages such as German, Dutch, Italian and Spanish. It has been reported that published studies on Arabic aphasia only comprised five studies, accounting for only 0.40% of the total literature on aphasia between 2000 and 2009 (Beveridge and Bak 2011). However, the selection of these studies was done from only 4 journals. There is a need for a comprehensive review of all studies to date on Arabic aphasia following PRISMA guidelines.

*What this study adds to the existing knowledge*

- This study reviewed 48 studies that presented original data on Arabic aphasia. We found that the majority of these studies were investigative in nature, where aphasic data was used to investigate linguistic or psycholinguistic theory. While these studies have clinical applications too, studies that developed protocols and materials for clinical assessment and tested the efficacy of various therapies and interventions in Arabic were comparatively limited.

*What are the clinical implications of this work?*

- Specific future directions are provided to aid in the development of assessments and interventions in service of Arabic‐speaking persons with aphasia.

## 1 Introduction

Aphasia refers to disturbances of both language production and comprehension because of damage to language processing areas in the cerebral cortex. These disturbances can be summarised in several clinical signs: lexical retrieval difficulties, fluency disorders, impaired repetition, auditory comprehension difficulties, compromised grammatical processing and reading and writing difficulties. Rather than failures of sensory systems, for example, hearing and vision, aphasia is a central impairment in which underlying language mechanisms are affected and can be a result of different types of lesions to the language centres in the brain.

The development of aphasia theory has benefitted from studies detailing the nature of language breakdown in different languages. The importance of cross‐linguistic investigation of aphasia is paramount due to the variation between natural languages in terms of phonology, morphology and syntax. Egia‐Zabala and Munarriz‐Ibarrola (2024) find that symptoms of aphasia manifest differently in different languages. Some languages are richer than others morphologically (e.g., English vs. German), resulting in different patterns of morpho‐syntactic errors in aphasia. Some languages have fixed word order, while others are more flexible (e.g., English vs. Arabic). These linguistic differences and the different error patterns they yield in aphasia contribute to a better understanding of the nature of language breakdown following aphasia and therefore have been the main driving force for cross‐linguistic investigations of patients with aphasia. To extend on linguistic variation, Arabic aphasia requires further scrutiny and development because of the stark differences between English and Arabic, with English as the language representing ‘89% of all papers’ (Beveridge and Bak 2011) on aphasia. For example, Ibrahim (2011) underlines Arabic's ‘special case of Diglossia,’ which is because of Modern Standard Arabic (MSA) and the many Arabic dialects. Ibrahim (2011) also justifies the deep differences in Arabic, as its forms ‘are phonologically, morphologically and syntactically different.’ An example of the difference is the phoneme pronunciation alteration from Classical Arabic according to the dialect, such as ‘ja’ being pronounced as ‘ga’ in the Egyptian dialect (Crutch et al. 2006).

Bates et al. (1991) conducted a review of cross‐linguistic studies of aphasia and concluded that investigating aspects of aphasia in different languages is important to verify whether findings based on English hold for other languages. An important principle to note is the fact that there is an ‘increasing number of aphasics in various regions of the world speaking their own native language,’ and the fact that assessment/diagnostic tools are only available in English creates a situation where ‘the variables impacting the outcome expand’ (Manaf and Shetty 2022). In addition, Manaf and Shetty (2022) also state how this ‘diagnostic scenario’ underlines ‘a major flaw in the method of service delivery.’ As Arabic is one of the most spoken languages internationally, there is a great demand and incentive to expand research and hone pathology services for Arabic aphasics.

Therefore, it has been postulated that broadening aphasia study to typologically different languages develops a better understanding of aphasia and underlying language mechanisms (e.g., Albustanji et al. 2013; Bates et al. 1991; Goral 2001; Khwaileh et al. 2015, 2017). While findings from aphasia have been mainly influenced by the structure of English, studies investigating aphasia in languages other than English have been undertaken in languages such as Dutch (Bastiaanse and Edwards 2004), German (Lorenz and Biedermann 2015), Hebrew (Bihovsky et al. 2024), Italian (Canu et al. 2020) and Spanish (Ardila 2001), among others. However, until relatively recently, research into aphasia in Arabic has been relatively limited. For example, Arabic, Hindi, Bengali, Russian and Portuguese were all ‘accounted for less than 0.5% of the aphasia literature’ (Beveridge and Bak 2011). The limited research is unfortunate, as it is one of the most widely spoken languages in the world (Lewis 2009), and typologically distant from Indo‐European languages that have been informing aphasia research for the past century. This warrants the need for a review of the available research on Arabic aphasia to identify areas in need of development and to guide future research endeavours. While the focus of the current review is studies with clinical applications, the review included studies with linguistic implications as well.

### 1.1 The Arabic Language

Arabic is the largest living member of the Semitic languages. It ranks fifth among world languages with respect to the number of speakers, after Chinese (Mandarin), English, Hindi and Spanish. It is an official language spoken in 23 countries extending from the Atlantic Ocean in the West to the Arabian Peninsula in the East. Arabic is also understood and read by the majority of Muslims (Lewis 2009). Furthermore, it has a rich morphological system, which contains morpho‐phonological and syntactic properties that could shed light on various aspects of aphasia, language processing and linguistic and psycholinguistic theories. Investigation of such properties would provide an insight into typical and atypical language processing, informing aphasia theory.

Typologically, Arabic contrasts with English on many features at different linguistic levels. At the phonological level, the two languages differ in the utilization of certain speech sounds, such as the pharyngealized sounds {t^ʕ^, d^ʕ^, ð^ʕ^, s^ʕ^} and the pharyngeals {ʕ, ħ} and the uvular {q} in Arabic. The phonotactics (permissible sound combinations and syllable structures) of the two languages are also different.

Furthermore, English words concatenate together in a linear order to produce a lexical item, such as in the word ‘compart‐ment‐al‐is‐ation.’ Therefore, omission of bound grammatical morphemes by aphasia patients in English could result in phonologically legal errors (e.g., ‘compartment’ or ‘mental’). However, in Semitic languages (e.g., Arabic and Hebrew), most words are formulated through a non‐linear system in which internal changes occur to a given word (McCarthy 1981). This system is based on several morphological units known as roots, vocalic patterns and CV skeletons. These units carry specific information concerning the semantic and grammatical nature of Semitic words. Thus, because of this non‐linear order of morphemes, omission errors could result in phonologically illegal utterances. For instance, the word /bana:t ‘girls’ consists of two bound morphemes, that is, the consonantal root /bnt/ and the CV skeleton /cvcv:c/. If the vocalic pattern is dropped because of an omission error, the resulting utterance will be phonologically illegal, that is, /bnt/.

Further to the difference in morpho‐phonology discussed above, Arabic adjectives must agree with the nouns they modify in terms of definiteness, number and gender. This is a morpho‐syntactic feature that does not exist in English. For example, the word /walad/ ‘boy’ is singular and masculine. To produce the phrase ‘tall boy’, the adjective ‘tall’ must agree with the noun to produce /walad t^ʕ^awi:l/ ‘a tall boy’. If the noun is replaced by a feminine, singular word such as ‘girl’, the adjective /t^ʕ^awi:l/ ‘tall’ must agree with the noun by adding the suffix ‐a. This suffix is a gender marker for singular, feminine nouns. The correct utterance would therefore be /bent t^ʕ^awi:l‐a/ ‘a tall girl.’

In some cases, what needs to be expressed in a whole sentence in English could be expressed in a single word in Arabic (i.e., /sa‐ju‐kallif‐u‐kum/ ‘it (masculine) will cost you (plural).’ Arabic differs from English in expressing definiteness of nouns. In English, an independent article (i.e., ‘the’) is required to precede the noun in question (i.e., ‘house’), creating a phrase consisting of two words: ‘the house.’ However, in Arabic, the definite article is simply attached to the noun (i.e., /bait/ ‘house,’ /l‐bait/) keeping the number of words in the phrase intact. Such words in Arabic carry several pieces of grammatical information (e.g., /l‐bait/ [definite, singular, masculine, noun] ‘the house’), while the grammatical information encoded in the single word ‘house’ in English does not exceed [singular, noun].

The difference between English and Arabic extends to syntax. Sentence formulation rules between the two languages differ at many levels; for example, word order of English is rigid compared to Arabic. In English, the default sentence structure is subject‐verb‐object (e.g., ‘The boy helped the girl’). Arabic, however, has a more flexible word order in which the verb can precede both the subject and object of the sentence (e.g., verb‐subject‐object) as in /sa:ʕad‐a al‐walad‐u al‐bent‐a/ ‘helped the boy the girl.’ The same sentence can also be expressed by other structures (e.g., /al‐walad‐u sa:ʕad‐a al‐bent‐a/ ‘The boy helped the girl.’ Additionally, sentence inflection in English is morphologically marked with at least a copula verb such as ‘is’ or ‘are’ (e.g., ‘The boy is tall’). On the other hand, Arabic has nominal sentences in which the sentence inflection is abstract and not morphologically marked (e.g., ‘al‐walad tawi:l’ noun‐adjective ‘the boy is tall’).

Despite these differences, research on Arabic aphasia was not available till the late 1990s. Nevertheless, literature on aphasia data from Arabic‐speakers has been growing in the last three decades (e.g., Boumaraf and Macoir 2015; Idrissi and Kehayia 2004; Prunet et al. 2000; Khwaileh 2011; Khwaileh et al. 2016, 2015, 2017, 2019, 2020; Mimouni et al. 1998; Mimouni and Jarema 1997), yet the number of studies on Arabic has been modest, which means that understanding of the nature of language breakdown in Arabic suffers from gaps. This issue extends to a poorer understanding of language processing and the nature of aphasia itself, as current models of language processing derive primarily from English aphasic data. Currently, speech language therapists for Arabic aphasics use ‘informal aphasia and/or translated western language assessments’ (Altaib et al. 2020), but the fact that Western and non‐Arab sources are ‘not sensitive to the linguistic and cultural features of the Arabic language’ underlines a major concern in getting ‘inaccurate diagnosis.’

Ultimately, the major differences between English and Arabic on the fundamental linguistic level underline the major concern and problematic nature of the overreliance on Western/English assessments and adapting and translating tools into Arabic. Arabic being significantly different from English on every linguistic level means there is little reason to continue referencing English frameworks, and instead, to work towards designing Arabic‐customised batteries, tools, frameworks, and so forth. The fact that Arabic aphasia research is such an underrepresented field with scarce contributions underlines how this field is lacking and needs research contributions and advancement to improve understanding and therapy schemes.

In addition, the addition of Arabic dialect variations and the sociolinguistic connections (different regions and societies affect different Arabic dialects further, on a semantic level) highlight a long‐term demand to also hone materials for respective dialects. Altogether, establishing how fundamentally different Arabic is from English creates a solid incentive and need to oversee the current research literature landscape.

## 2 Aim

This paper provides a systematic review of English‐language publications reporting data on Arabic‐speaking aphasics. The goal is to provide an overview of the body of Arabic aphasia literature and critically assess said body. We aimed to examine the current clinical and linguistic scope and the limitations. Through pinpointing gaps in literature and identifying research inconsistencies, this review seeks to provide a helpful overview and inform future research directions. Since Arabic aphasia research is a niche field, the scope of this review is intentionally broad to accommodate all relevant findings without forming strict criteria on an already small research area.

## 3 Methodology

The methodology of the systematic review was conducted in accordance with the protocol of the Preferred Reporting Items for Systematic Reviews and Meta‐Analyses (PRISMA) to ensure thoroughness. As Arabic aphasia research is a niche, the methodology scope was designed to prioritize replicability and clarity so that future researchers can effectively build upon this review to contribute further research and development into treatment of Arabic persons with aphasia. The following sections describe the steps carried out in the methodology:

### 3.1 Systematic Review's Strategy

**FIGURE 1:**
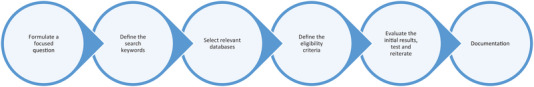
The review strategy.

Given the interdisciplinary nature of this review scope, the strategy had to be both detailed and succinct to ensure that the research process and analyses were always relevant, efficient and objective. The review strategy (see Figure 1 below) started with the formulation of a solid research objective of systematically compiling relevant research about the underrepresented sub‐field of Arabic aphasia. Search keywords were selected next; they would later yield the relevant publications systematically. Afterwards, relevant databases were selected, from which the systematic review extraction would take place. The eligibility criteria were then defined to ensure quality assurance of the research data, and after, two levels of research data screening would occur (initial and reiteration). Finally, we processed the data findings and documented them.

### 3.2 Research Question and Aim

The research aim was to conduct a thorough, expansive and systematic review of all published research about Arabic aphasia. We sought to identify and analyse the status of this research sub‐field to highlight the significance and relevancy it contributes.

The specific research questions are:

1. What is the current state of available clinical resources for assessment and therapy/intervention?
2. What is the current theoretical understanding of the ways in which aphasia manifests in such a typologically distinct language, considering Arabic's unique morphosyntax?

### 3.3 Keywords

Fourteen words were selected: ‘Anomia, Anomic, Agrammatism, Broca's, Conduction, Expressive, Fluent, Global, Mixed transcortical, Non‐fluent, Receptive, Transcortical motor, Transcortical sensory, Wernicke's’. The search keywords were selected on the basis that they represent the core, scientific classifications of aphasia. Also, to ensure objectivity and efficiency, both ‘Arabic’ and ‘aphasia’ accompanied every search word. The year of publication was not specified in the search, as the aim of the review is to identify all available literature published in an under‐researched field.

### 3.4 Databases and Sources

Relevant databases and search key terms were identified prior to the review. To serve the purposes of this review, five online bibliographic databases, APA PsycINFO, PubMed, PubPsych, ScienceDirect, and Springer Nature Link (see Table 1), were identified based on their specialized general field, and their subject relevance to aphasia, Linguistics, Psychology, Medical and Health Sciences, Medicine, Behavioural Sciences, Cognitive Science, Neuroscience, Rehabilitation, and Speech and Language Pathology.

**TABLE 1:** List of major sources used in the review.

Source	Type of source	Description
APA PsycINFO	Academic database	Produced by the American Psychological Association, APA PsycINFO has 5 000 000 interdisciplinary, peer‐reviewed records. APA PsycINFO possesses research of behavioural and social science research.
Google Scholar	Search engine	Freely accessible and has an expansive scope due to a wide index of literature. Due to the risk of quality variation, all papers sourced from Google Scholar were screened on Scimago.
PubMed	Academic database	PubMed comprises more than 30 million citations about science and biomedical literature.
PubPsych	Academic database	PUBPSYCH is an open‐access platform for psychological resources, offering a comprehensive and balanced selection of resources.
ScienceDirect	Academic database	Hosting more than 4000 journals, ScienceDirect specializes in providing scientific and medical literature.
Springer Nature Link	Academic database	Springer Nature Link contains a vast online collection of research in the medical, technological and scientific fields.

As there are no readily available online databases for Arabic publications, all five databases used are Western/European‐based meaning that all research papers handled in this systematic review are in English. Besides their breadth of subject/disciplinary coverage, the databases were all also chosen for their international, mainstream status. Since Arabic aphasia research is already underrepresented, strategically selecting internationally known and mainstream databases strongly ensured that we would be able to find as much relevant information as possible, while still maintaining methodological rigour and impartiality.

In addition to academic databases, Google Scholar was also included in this systematic review in order to ensure that a comprehensive overview of research was included. As the five selected academic databases were all internationally renowned, highly competitive and Western‐based, many relevant studies may not be indexed on such platforms. Given the fragmented foundations of Arabic aphasia research and its underrepresentation, the likelihood of relevant literature being independent of mainstream academic databases is much higher. Since Google Scholar is a search engine focusing on scholarly work, it offers important contributions that may have otherwise been inaccessible, in the form of grey literature.

According to PRISMA's guidelines, the incorporation of grey literature can enhance the inclusivity of a systematic review, which ensures a more representative overview. A comprehensive, wide overview is essential to this research's objective because prior to identifying and suggesting new frameworks and techniques, the research status quo must be fully grasped. Only then can limitations be pinpointed. Quality assurance of literature and research systematicity while using Google Scholar is covered in the following section, ‘Eligibility Criteria.’

### 3.5 Eligibility Criteria

The following eligibility criteria were applied:

- The final list of publications had to be reporting and/or analysing clinical or linguistic data from native Arabic‐speakers with aphasia (including all Arabic dialects).
- They must be published in English.
- If the paper was derived from Google Scholar, ensure they were published in journals indexed in SCImago Journal Rank (SJR).

### 3.6 Evaluation

The evaluation stage involved sorting through the yielded articles, filtering them against the eligibility criteria mentioned above, and assessing the process based on the search outcome. The motivation for the evaluation stage was to manage the risk of bias in selecting studies and to ensure quality assurance of literature. In the evaluation stage, two independent reviewers approved each paper's Level 1 and Level 2 verification. Both reviewers had backgrounds in linguistics (minimum qualification being undergraduate level, maximum qualification being masters’).

Once the search was conducted and the eligibility criteria were applied, the initial results were evaluated. The evaluation of the initial results was done in relation to the research objective and at two levels; Level 1 was the initial filtering stage which included reading the title and abstract of yielded publications, while Level 2 (including all papers that passed Level 1 stage) involved full article reading, and documenting the article findings.

### 3.7 Documentation

The documentation protocol involved two documents. The first was a table log of all searches, dates of search and keywords used. The second document was an extended table that was completed for the total papers that passed Level 2 filtering. The second table included the main elements of any given study in the field (study title, study objective, theoretical framework, the Arabic dialect investigated, main findings and recommendations).

Studies filtered from the search were based on two factors: (1) Purpose – (Investigative, Assessment or Therapy); (2) Framework – (Clinical, Neurolinguistic, Psycholinguistic). The reasoning for purpose being sorted into Investigative, Assessment, and Therapy is to accommodate the general overview of research purpose for Arabic aphasia literature. Assessment and Therapy papers represent the more clinical, case‐study based and treatment‐based papers. To be specific, Therapy focuses on the actual rehabilitation and treatment strategies and innovations whereas Assessment focuses on adaptation or evaluation of existing diagnostic tools and batteries. Investigative represents papers that focus more on analysis and exploration of frameworks or linguistic models in reference to aphasia. The Framework is to establish the type of subject discipline the paper focuses on.

## 4 Results

**FIGURE 2:**
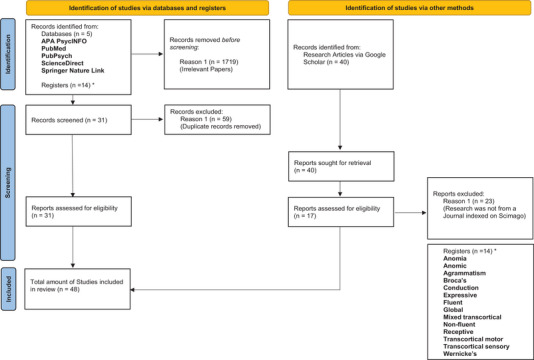
Selection of studies as per PRISMA guidelines.

The search process yielded 48 studies, of which 31 studies were compiled from the traditional systematic review from academic databases. The remaining 17 were derived from Google Scholar after ensuring the paper's journal reliability on Scimago. Figure 2 below reports the selection process as per PRISMA guidelines.

The studies were published between 1997 and 2024. Figure 2 shows the number of studies published on Arabic aphasia per year. These studies combined reported on the aetiology of the cases to be predominantly cerebrovascular accidents (CVA). There was one case of primary progressive aphasia (PPA) (Table 2).

**TABLE 2:** Breakdown of aphasia types covered.

Aphasia Type	*n*
Broca's aphasia	18
Wernicke's aphasia	7
Global aphasia	6
Conduction	5
Anomia	5
Transcortical motor	4
Transcortical sensory	3
Fluent	2
Primary progressive aphasia (PPA)	1

The review covered a total of 51 studies of various aphasia types. The largest portion, at 37.3% (*n* = 19), were papers that did not specify an aphasia type. Meanwhile, Broca's aphasia was the most studied type, representing 35.3% (*n* = 18). Next, Wernicke's followed at 13.7% (*n* = 7). Global (11.8%), while anomia and conduction (both 9.8%, respectively) all received similar moderate attention. Finally, the least investigated and mentioned types were transcortical motor aphasia (7.8%), transcortical sensory aphasia (5.9%), and fluent aphasia (3.9%). Finally, the least studied type was PPA, with only one study focusing on it.

The data also revealed that certain varieties within the Arabic‐speaking world received more attention from researchers than others. See Table 3 for details about Arabic dialects covered. In terms of dialects, a significant amount did not specify studying a specific dialect or the MSA, and this was 44.2% (*n* = 19). There was a total of nine dialects mentioned, as well as MSA. Among the dialects, the most representation was equal between Jordanian, Palestinian, and Saudi Arabic dialects—they all had an amount of 11.6% (*n* = 5). The least studied dialects included Algerian Arabic (4.7%), Lebanese Arabic (2.3%), Gulf Arabic (2.3%), and Iraqi Arabic (2.3%).

**TABLE 3:** Breakdown of Arabic dialects covered.

Dialect	Number of papers
NA (not available)	19
Jordanian Arabic	5
Palestinian Arabic	5
Saudi Arabic	5
Egyptian Arabic	4
Moroccan Arabic	3
Modern Standard Arabic	2
Algerian Arabic	2
Lebanese Arabic	1
Gulf Arabic	1
Iraqi Arabic	1

Through the chronology (see Table 4), we can observe that prior to 2011 (only 13 years ago), there were significantly limited amounts of papers, not even reaching 10 papers. Within the second decade of the 21st century (2011–2020), we can observe a healthy increase to 19 papers. The growing amount of research in Arabic aphasia continues, as the next 4 years (2021–2024) foresaw that the same number of papers (19) as the decade prior had been found.

**TABLE 4:** Chronological distribution of papers.

Year	Number of papers
1997–2000	2
2001–2010	8
2011–2020	19
2021–2024	19

A detailed overview of all the studies included in this review along with the classification of each as per purpose, framework, dialect, linguistic level, and language dialect is presented in Table 5.

**TABLE 5:** Overview of studies reviewed.

No	Title	Aphasia type	Purpose	Framework	Dialect
1	Abou El‐Ella et al. (2013). Modification and Standardisation of the Arabic Version of the Comprehensive Aphasia Test.	NA	Assessment	Psycholinguistics, Clinical	Egyptian Arabic
2	Abou‐Elsaad et al. (2018). Developing an Arabic Screening Test for Adult‐Onset Chronic Aphasia.	NA	Assessment	Clinical, Psycholinguistics	Egyptian Arabic
3	Adam, H. (2014). Dysprosody in aphasia: An Acoustic Analysis Evidence From Palestinian Arabic	Broca's	Investigative	Neurolinguistics, Psycholinguistics	Palestinian Arabic
4	Albustanji et al. (2013). Agrammatism in Jordanian‐Arabic‐Speakers.	Broca's	Investigative	Clinical, Psycholinguistics	Jordanian Arabic
5	Aldera, M. (2017). Application of the Dual‐Route Model in Exploring Dyslexia and Dysgraphia in Arabic‐Speaking Adults With Aphasia: Clinical and Theoretical Implications.	Broca's, Transcortical Motor, Anomia, Conduction, Wernicke's	Investigative	Psycholinguistics, Neurolinguistics Clinical	Modern Standard Arabic
6	Alduais, A. M. S. (2013). Identifying Typical and Atypical Manifestations of Pragmatic Language Impairment in Arabic: A Multi‐Method Study of individuals With Developmental Dysphasia.	NA	Assessment	Neurolinguistics, Psycholinguistics, Clinical	NA
7	Alloush et al. (2024). Aphasia Outcome: The Role of Diffusion Tensor Tractography in Patients With Acute Ischemic Stroke.	Broca's, Wernicke's, Global, Transcortical motor, Transcortical sensory, Conductive	Investigative	Clinical, Neurolinguistics	NA
8	Alohali, N. (2022). Extending the Scope of Sentence Therapy in Aphasia Across English and Arabic.	NA	Assessment	Clinical, Psycholinguistics	Saudi Arabic
9	Alqhazo, M., and E. Abu Jaleel (2024). Subject‐Verb Agreement and Tense in Jordanian Arabic Broca's Aphasia.	Broca's	Assessment	Psycholinguistics	Jordanian Arabic
10	Al‐Shdifat et al. (2018). Exploring the Efficacy of Melodic Intonation Therapy With Broca's Aphasia in Arabic.	Broca's	Therapy	Clinical, Psycholinguistics	Jordanian Arabic
11	Altaib et al. (2020). From Informal to Formal: The Preliminary Psychometric Evaluation of the Short Aphasia Test for Gulf Arabic‐Speakers (SATG).	NA	Assessment	Psycholinguistics, Clinical	Saudi Arabic
12	Al‐Thalaya et al. (2017). Reliability and Validity of Bedside Version of Arabic Diagnostic Aphasia Battery (A‐DAB‐1) for Lebanese Individuals.	NA	Assessment	Psycholinguistics, Clinical	Lebanese Arabic
13	Alyahya, R. S. W., and J. Druks (2015). The adaptation of the Object and Action Naming Battery Into Saudi Arabic.	NA	Assessment	Clinical, Psycholinguistics, Sociolinguistics	Saudi Arabic
14	Alyahya, R. S. W. (2024b). The public Awareness and Knowledge of Aphasia in Saudi Arabia.	NA	Investigative	Sociolinguistics	Saudi Arabic
15	Alyahya, R. S. W. (2024a). The Development of a Novel, Standardized, Norm‐Referenced Arabic Discourse Assessment Tool (ADAT), Including an Examination of Psychometric Properties of Discourse Measures in Aphasia.	NA	Assessment	Clinical, Psycholinguistics	Saudi Arabic
16	Basura et al. (2024). Feasibility Study of the Boston Naming Test for the Arab Population.	NA	Assessment	Sociolinguistics, Psycholinguistics	NA
17	Beveridge, M. E. L., and T. H. Bak (2011). The Languages of Aphasia Research: Bias and Diversity.	NA	Investigative	Sociolinguistics, Psycholinguistics	NA
18	Boumaraf and Macoir (2015). The Influence of Visual Word Form in Reading: Single Case Study OF An Arabic Patient With Deep Dyslexia.	Broca's	Investigative	Psycholinguistics	Modern Standard Arabic
19	Crutch et al. (2006). The Different Frameworks Underlying Abstract and Concrete Knowledge: Evidence From a Bilingual Patient With a Semantic Refractory Access Dysphasia.	NA	Investigative	Clinical, Psycholinguistics	Iraqi dialect
20	Dakwar et al. (2018). Diglossic Aphasia and the Adaptation of the Bilingual Aphasia Test to Palestinian Arabic and Modern Standard Arabic.	NA	Assessment	Sociolinguistics, Psycholinguistics	Palestinian Arabic
21	Elhakeem et al. (2021). Post‐Stroke Aphasia Rehabilitation Using a Computer‐Based Arabic Software Program: A Randomized Controlled Trial.	Broca's, Anomia, Global, TCM, Mixed	Therapy	Clinical	Egyptian Arabic
22	Elmongui et al. (2022). Diffusion Tensor Imaging of Dorsal Stream Language Areas in patients With Post‐Stroke Aphasia.	Broca's, Wernick's	Assessment	Clinical, Neurolinguistics	NA
23	El Ouardi et al. (2023). The Moroccan Arabic Bedside Western Aphasia Battery‐Revised: Linguistic and Psychometric Properties.	NA	Assessment	Psycholinguistics, Clinical	Moroccan Arabic
24	El‐Tallawy et al. (2019). Relative Frequency and Prognosis of Vascular Aphasia (Follow‐Up at 3 Months) in the Neurology Department of Assiut University Hospital.	Global, Broca's, Wernicke's, Conduction, Mixed Transcortical, Transcortical motor, Transcortical sensory, Anomia	Assessment	Clinical, Psycholinguistics	NA
25	Fahmy and Elshebawy (2021). Effect of High Frequency Transcranial Magnetic Stimulation on Recovery of Chronic Post‐Stroke Aphasia.	NA	Therapy	Clinical, Neurolinguistic	NA
26	Faroqi‐Shah and Waked (2010). Grammatical Category Dissociation in Multilingual Aphasia.	NA	Assessment	Psycholinguistics	NA
27	Friedmann (2001). Agrammatism and the Psychological Reality of the Syntactic Tree.	Broca's	Investigative	Psycholinguistics	Palestinian Arabic
28	Friedmann (2002). Question Production in Agrammatism: The Tree Pruning Hypothesis.	Broca's	Investigative	Psycholinguistic	Palestinian Arabic
29	Hafiz et al. (2023). Construction of Structured Arabic Aphasia Caregiver Guide and Studying Its Effectiveness in Improving Caregivers' Awareness and Communication With Their Aphasic Patients.	NA	Therapy	Clinical, Psycholinguistic	NA
30	Hisham (2021). Vowel Production in Aphasia: Preliminary Acoustic Findings From Arabic.	Broca's	Assessment	Psycholinguistic	Palestinian Arabic
31	Ibrahim (2008). Performance in L1 and L2 Observed in Arabic‐Hebrew Bilingual Aphasic Following Brain Tumor: A Case Constitutes Double Dissociation.	NA	Investigative	Psycholinguistics, sociolinguistics	NA
32	Ibrahim (2009). Selective Deficit of Second Language: A Case Study of a Brain‐Damaged Arabic‐Hebrew Bilingual Patient.	NA	Investigative	Neurolinguistics, Psycholinguistics	NA
33	Ibrahim (2011). Neurocognitive Aspects of Processing Arabic and Hebrew.	NA	Investigative	Psycholinguistics	NA
34	Jomaa et al. (2022). An Egyptian Patient Story: Multilingual Role in Post‐Stroke Aphasia Recovery.	Global, Conduction	Investigative	Clinical, Neurolinguistic	Cairene Dialect (Egyptian)
35	Khedr et al. (2020). A Hospital‐Based Study of Post‐Stroke Aphasia: Frequency, Risk Factors, and Topographic Representation.	Broca's, Anomia, Transcortical motor, Wernicke's, Transcortical sensory, Conduction, Global.	Assessment	Clinical, Neurolinguistics	Egyptian Arabic
36	Khwaileh et al. (2015). Morpho‐Syntactic Processing of Arabic Plurals After Aphasia: Dissecting Lexical Meaning From Morpho‐Syntax Within Word Boundaries.	Anomia	Investigative	Psycholinguistic	Jordanian Arabic
37	Khwaileh et al. (2017). Lexical Retrieval After Arabic Aphasia: Syntactic Access and Predictors of Spoken Naming.	Anomia	Investigative	Psycholinguistics, Clinical	Jordanian Arabic
38	Khwaileh et al. (2020). A Linguistically‐Driven Response Categorisation Protocol for Arabic Nouns and Verbs: Clinical and Research Applications.	NA	Assessment	Clinical, Psycholinguistic	Gulf Arabic
39	Knoph (2013). Language Intervention in Arabic–English Bilingual Aphasia: A Case Study.	NA	Therapy	Psycholinguistics, Clinical	Palestinian Arabic
40	Mimouni, Z., and G. Jarema (1997). Agrammatic Aphasia in Arabic.	Broca's	Investigative	Psycholinguistics	Algerian Arabic
41	Mimouni et al. (1998). The Mental Representation of Singular and Plural Nouns in Algerian Arabic as Revealed Through Auditory Priming in Agrammatic Aphasic Patients.	Broca's	Investigative	Psycholinguistics	Algerian Arabic
42	Qorchi, B., and A. Bouchara (2017). Agrammatism and Other Aphasia‐Related Disorders in Moroccan Arabic‐Speaking Aphasics.	Broca's, Wernicke's, Global	Investigative	Psycholinguistics, Clinical	Moroccan Arabic
43	Rami et al. (2024). The adaptation of the Object and Action Naming Battery Into Moroccan Arabic: Norms for Name Agreement, Frequency, Imageability, Visual Complexity, and Age of Acquisition.	fluent	Assessment	Psycholinguistic	Moroccan Arabic
44	Summaka et al. (2023). Computed Tomography Findings as Early Predictors of Long‐Term Language Impairment in Patients With Traumatic Brain Injury.	NA‐Does not specify	Assessment	Neurolinguistic	NA‐Does not specify
45	Taiebine, M., and M. El Alaoui Faris (2019). Neurolinguistic Analysis of a Case of Phonological Alexia in Arabic Language.	Broca's	Investigative	Psycholinguistic, Neurolinguistic	NA
46	Taiebine, M., and M. El Alaoui Faris (2023). Atypical Mixed Logopenic and Semantic Progressive Primary Aphasia (PPA) in Arabic With White Matter Abnormalities.	Primary progressive aphasia (PPA)	Investigative	Clinical, Neurolinguistic	NA
47	Tavano et al. (2009). Language and Cognition IN A Bilingual Child After Traumatic Brain Injury in Infancy: Long‐Term Plasticity and Vulnerability.	Fluent	Investigative	Clinical, Psycholinguistic	NA
48	Vajramani et al. (2008). Bilingual Aphasia Due to Spontaneous Acute Subdural Haematoma From a Ruptured Intracranial Infectious Aneurysm.	NA	Investigative	Clinical, Neurolinguistic	NA

Overall, the above results demonstrate that certain areas within the aphasia body of data from Arabic‐speakers are still sparse, warranting this comprehensive review and further studies to fill the gaps in the literature. While there has been substantial growth in data from Arabic, particularly from 2013 to date, the field is still considered underdeveloped when compared to the status and number of speakers of the Arabic language, compared to other well‐studied languages in Europe.

The following paragraphs will delve into the content of each study reviewed and present detailed results in a thematic approach, that is, grouped under assessment studies, therapy studies, and investigative studies.

### 4.1 Assessment Studies

The overarching frameworks used across assessment studies are included for analysis. The current review showed that until 2016, the development of aphasia assessment tools for the Arabic language has been restricted to translations and adaptations from tests developed for other languages. These included the Egyptian Arabic version of the Comprehensive Aphasia Test (for Egyptian Arabic: Abou El‐Ella et al. 2013), non‐standardized translations of the Boston Diagnostic Aphasia Examination (various Arabic dialects: Goodglass and Kaplan 1983), the Object and Action Naming Battery (for Saudi Arabic: Alyahya and Druks 2015). Adapted aphasia tests and batteries’ development were mostly successful, and they were shaped/adapted for effectiveness and accuracy by the cultural, pragmatic and sociolinguistic features of the Arab‐speaking world, unlike tools that were only translated.

In addition, the Bilingual Aphasia Test (BAT) has been adapted to several Arabic dialects (e.g., Paradis and El Halees 1989; Arabic version). The BAT is different from all the tests translated above; it is designed to assess bilinguals using language pairs, and it has been adapted for over 60 languages, including Arabic. Recently, Dakwar et al. (2018) adopted the BAT to develop a test for the diglossic situation in Palestine (MSA) and Palestinian Arabic (PA). The authors highlight the structural differences between the Palestinian dialect and MSA, as they are evident in all linguistic levels, including phonology, lexical semantics, and morpho‐syntax. Dakwar et al. (2018) followed the guidelines published by Paradis and Libben (1987), which provide an outline of the different sections of the BAT, how to select items for the assessment, and methods for addressing cross‐linguistic considerations. While the Arabic BAT is not a direct translation of the original BAT, the stimuli selected are not matched for psycholinguistic variables affecting language processing for Arabic, such as frequency, name agreement, image agreement, visual complexity, age of acquisition, familiarity, and healthy naming latencies. This is also the case for the translated tests mentioned above.

Alduais (2013) led a multi‐method study to assess the degree of usability of an Arabic version of the Test of Pragmatic Language‐2 (TOPL‐2) in terms of identifying pragmatic language impairment (PLI) in people with developmental dysphasia (DD). The findings indicate how TOPL‐2 was a valuable psycholinguistic tool. Cultural adaptation and the need for ‘Arabization’ were integral, such as replacing foreign names like ‘Cindy’ and ‘Brad’ with Arab names ‘Fatima’ and ‘Badr,’ respectively. A large obstacle was the adaptation of the picture book, which represented Western culture, ‘featuring girls dressed in a style that is not common in Arabian countries.’ Similarly, Alohali's (2022) thesis presents the development of Arabic language assessment and therapy materials for aphasics, evaluating the challenges and successes of the adaptation of Western tools in light of socio‐cultural differences and contexts. Furthermore, Basura et al. (2024) sought to analyse and evaluate the usability and cultural biases of the Boston Naming Test (BNT‐60) as opposed to making an adapted Arabic version. Using BNT‐60 and Montreal Cognitive Assessment (MOCA) on healthy native Saudi Arabic‐speaking students showed that they had very lower accuracy to US norms on culturally unfamiliar items, underlining proof of cultural bias and need of a test adaptation.

Al‐Thalaya et al. (2017) sought to develop the Arabic Diagnostic Aphasia Battery (A‐DAB‐1) by adapting the Western Aphasia Battery‐Revised (WAB‐R). Cultural and linguistic details were adapted appropriately using the Delphi method. Tested on 60 healthy and 30 stroke patients, results showed success. Similarly, El Ouardi et al. (2023) adapted the Bedside Western Aphasia Battery–Revised (WAB‐R) into Moroccan Arabic (MA) with linguistic and cultural constraints successfully. The Bedside MA‐WAB‐R psychometric properties were evaluated, and it became the first standardized aphasia assessment tool for Moroccan Arabs. Furthermore, Rami et al. (2024), studying Moroccan Arabic‐speakers, used the Object and Action Naming Battery (OANB) in order to identify and establish normative features for a Moroccan Arabic version of a naming battery. Additionally, Alyahya (2024b) developed the Arabic Discourse Assessment Tool (ADAT) for Arabic‐speaking aphasics. ADAT is standardized and culturally appropriate for Arab aphasics, an important distinction given the major existing literature's assessment tools are all Western‐based.

Alqhazo and Abu Jaleel (2024) studied the types of deficits Jordanian Arabic‐speaking aphasics may have in regard to tense and agreement. Findings show that there is a selective deficit in the production of past tense forms, linking it to past hypotheses about agrammatism in Arab speakers. In a similar vein, Hisham (2021) sought to examine vowel production in Palestinian Arabic aphasics suffering from Broca's aphasia. Findings aligned with past research that demonstrated abnormal vowel production in Broca's aphasia. Additionally, Faroqi‐Shah and Waked (2010) studied a trilingual aphasic's grammatical category dissociation and discovered that despite semantic processing and comprehension staying mostly intact, the patient had noun‐verb dissociation and severe verb production impairment, meaning their deficit was likely nonsemantic. Findings support that a word's grammatical category shares its information across multilingual lexical organization.

A hospital study by Khedr et al. (2020) investigated the frequency, risk factors, and lesion localization of post‐stroke Arabic aphasics. They discovered that global and motor aphasia were the most common types and that there was a ‘wide degree of overlap between the distribution pattern of brain damage and the aphasia type.’ Similarly, El‐Tallawy et al. (2019) analysed post‐stroke aphasia in terms of its clinical types, prognostic factors, and its prevalence across 1508 stroke patients. Of these, 107 had PSA, with global aphasia being the most common subtype at 66.4%, and finally, prognostic factors were cerebral haemorrhage, subcortical aphasia, smaller lesion size, and younger age. Additionally, Summaka et al. (2023) found out that left hemisphere brain injuries can cause the most severe language deficits. Also, they were able to conclude how a higher Helsinki CT score, meaning greater brain damage, was linked to worse language outcomes.

Finally, Elmongui et al. (2022) investigated post‐stroke aphasics through diffusion tensor imaging (DTI) and were able to successfully conclude that DTI has potential as a non‐invasive method for assessing post‐stroke aphasics' language areas.

The field has seen an increase of papers reporting various tests being developed specifically for the Arabic language for various dialects (Qatari Arabic: Khwaileh et al. 2016; Jordanian Arabic: AlSwaiti 2019; Saudi Arabic: Aseeri 2018 and Altaib et al. 2020; Egyptian Arabic: Abou‐Elsaad et al. 2018) since 2016. These tests are not all comprehensive batteries. Abou‐Elsaad et al. (2018)’s test is a quick screening test for adult‐onset chronic aphasia that provides information about the type and the severity of aphasia, and it is based on the syndrome approach rather than building a comprehensive neuropsychological profile of the tested patient. The test takes less than 20 min to complete. AlSawiti (2019) reports on the content validity of a test developed for Jordanian‐Arabic‐speakers with aphasia. The author does not report on any patient or normative data, and states that these remain as future plans for this test. Altaib et al. (2020) describe the preliminary development and the preliminary psychometric evaluation of the short aphasia test for Gulf Arabic. The test is not a comprehensive test and is still awaiting further validation. This is the same case for the test reported in Aseeri (2018).

In addition to the tests mentioned above, through the review of unindexed journals in the Arabic‐speaking world, the authors came across a test called the Kasr El‐Aini Arabic Aphasia Test‐KAAT (for Egyptian Arabic: Hassanein et al. 2002). The authors of the test were contacted to obtain further information about the test; a response is still awaited. Most of these tests are works in progress, and the actual tests were not found to be published anywhere. The first author of the current study contacted some of the authors mentioned above via email to find out more about these tests, and it was confirmed that most of these tests are still awaiting validity and reliability data.

With the exception of Khwaileh et al. (2016), all the above‐mentioned tests are not informed by recent psycholinguistic findings from the Arabic language. For example, none of the modified, translated, and Arabic tests described above have been controlled for variables such as imageability, age of acquisition, frequency, familiarity, length, and name agreement. Khwaileh et al. (2016) describe four phases for the development of their Qatari Arabic aphasia test: a comprehensive review of Arabic linguistics and psycholinguistics; a linguistically informed normative database development; the development of a comprehensive set of aphasia subtests and validation of the subtests with control participants and patients with aphasia. All the stimuli used in their test are controlled for the visual complexity of their pictures, image agreement, imageability, age of acquisition, frequency, familiarity, phonemic and syllabic length, name agreement, and normative naming latency. This agrees with the roadmap proposed by Khwaileh and Grosvald (2019), in which they discuss the development phases of assessments for Arabic‐speakers following CVA.

### 4.2 Therapy Studies

Clinical intervention studies on Arabic aphasia have also been limited. To the best of the authors’ knowledge and according to the review carried out, only five studies have been published addressing intervention. Firstly, an example is Knoph (2013), who published ‘Language intervention in Arabic–English bilingual aphasia: A case study’, in which the author reports on a bilingual speaker of Arabic with chronic moderate to severe non‐fluent aphasia. The purpose of the study was to determine whether the treatment of verb production in the L2 of a bilingual Arabic–English speaker with moderate to severe chronic aphasia would lead to improvements in both the treated L2 and the untreated L1 (cross‐language transfer). Patient AF was 62 years old, a right‐handed male diagnosed with chronic moderate to severe non‐fluent receptive and expressive aphasia following a left hemisphere stroke at the age of 61. AF was a trilingual speaker of Levantine Arabic as his L1 and, English and German as his L2 and L3 languages. AF was assessed using the Jordanian Arabic version of the BAT (Paradis and El Halees 1989).

Therapy was conducted in the participant's L2 (English), to establish whether treatment in one language would result in transfer to the other L1 (Arabic). The results of the BAT for post‐treatment of English indicated that there was a significant improvement in the performance of English, with improvement in auditory discrimination, grammaticality judgement, and semantic opposites. For the untreated L1 (Arabic), the BAT results indicated a significant improvement in performance as well. Particularly in complex commands, semantic categories, antonyms, semantic acceptability, and listening comprehension. For the linguistic clusters of English, the BAT scores indicated that there was an improvement at all linguistic levels (phonology, syntax, semantics) except for morphology. For the untreated Arabic, overall results indicated an improvement in one main level—semantics. However, there was a treatment generalization with syntax, phonology, and morphology. Overall, AF exhibited improvement in his treated L2 (English) and subsequent transfer to his L1 (Arabic) but not in all linguistic levels. Improvement was mainly in semantics, syntax, and, to an extent, lexical retrieval, but not in the phonological level. The results of language transfer of the treatment of L2 to L1 of the current study were in line with Kroll and Stewart's (1994) RHM model. The results contribute to the theory that there is a shared conceptual system for L1 and L2, as there was a significant improvement in the lexical‐semantic level. AF's improvement in the treated L2 and thus the transfer of L1 is in line with Edmonds and Kiran (2006), and Faroqi‐Shah et al. (2010), who suggested that training the less‐dominant language can result in cross‐language transfer in an unbalanced bilingual, leading to improvement in the dominant language.

Another therapy study, this time by Fahmy and Elshebawy (2021) analysed the effects of excitatory repetitive transcranial magnetic stimulation (rTMS) on the recovery of chronic post‐stroke aphasia patients. 10‐Hz rTMS was applied over the Broca's area for 10 sessions, with assessment being done before, immediately after, and 1 month after, through two tests: the Aphasia Severity Rating Scale (ASRS) and Kasr Al‐Aini Arabic Aphasia Test (KAAT). Successful results demonstrated that significant improvement was seen across overall language skills as well as aphasia severity, proving both short‐ and long‐term effectiveness and viability.

Thirdly, Hafiz et al. (2023) sought to develop and evaluate an Arabic Aphasia Caregiver Guide through testing two groups: Group 1 (Cases group) had conventional language therapy and caregiver training/education, and Group 2 (Control group) got conventional therapy only. Results showed how Group 1 had significantly better outcomes in terms of caregiver awareness and language outcomes. Despite having less of a linguistic base, it is an important contribution to the review to bring awareness to Arab communities.

A fourth therapy study on Arabic was carried out by Al‐Shdifat et al. (2018). The authors report on the efficacy of melodic intonation therapy in a Jordanian Arabic‐speaker (patient MK) with Broca's aphasia. The authors describe an 8‐week (90 min; 6 days a week) therapy programme using melodic intonation therapy after establishing multiple baseline designs across automatic and self‐generated phrases. The authors report that MK improved his expressive productions post‐treatment in automatic (75% accuracy criterion) and self‐generated phrases (reached criterion and remained constant at follow‐up). The authors conclude that melodic intonation therapy is a viable option for Jordanian Arabic‐speaking patients with Broca's aphasia.

A fifth intervention study on Arabic was a post‐stroke aphasia rehabilitation study using a computer‐based Arabic software programme (Elhakeem et al. 2021). The aim of the study was to assess the effectiveness of Arabic aphasia therapy using computer‐based programmes, and to compare it to the conventional language therapy programmes. They used a randomised controlled trial with blinded endpoint evaluation with 50 aphasic patients (group A and group B), receiving 48 sessions using computer‐based therapy with group A, and the conventional therapy with group B. The authors used the translated Boston Diagnostic Aphasia Examination to measure therapy outcomes. Results showed a significant improvement from the baseline in both groups. There was no significant difference in the post‐therapy results between different groups, suggesting that the computer‐based Arabic software programme was as effective as the conventional therapy programme in the improvement of language abilities in Arabic aphasia.

### 4.3 Investigative Studies

This subsection provides insight about investigative research in Arabic aphasia. Taking into account the fact that most of the studies published on Arabic aphasia are driven by linguistic motivations rather than clinical drives, the primary nature of research for Investigative studies focuses on a psycholinguistic or linguistic lens, rather than clinical as seen prior to this.

The review found that the most common linguistic level investigated was morphology. Morphological properties of Arabic are the most distinct features of the language in comparison to English and other Indo‐European languages. Existing theories which have been proposed to explain the formulation of morphemes in Semitic languages have been tested against aphasic data. Findings from the studies in the current review provide evidence in support of the root‐pattern theory proposed by McCarthy and colleagues (1981, 1990), in which they claim that Arabic words are made of at least two bound morphemes: a root and a vocalic pattern. The root carries semantic information, whereas the vocalic pattern carries derivational information. There are currently no models of morphological composition which take into account this type of non‐concatenative formulation of morphemes and segments, as current models are mainly based on English and German (Clahsen 1999, 2006; Pinker 1999; Pinker and Ullman 2002; Rumelhart and McClelland n.d.; Stockall and Marantz 2006).

The findings of Ibrahim (2008), who studied a bilingual aphasic (L1 Arabic and L2 Hebrew), support that multilinguals have differential neural representation of languages in the brain, even for languages that are closely related/similar. In addition, Ibrahim (2009) investigated how bilingual aphasics' level of language impairment can be of different levels and have different sources. The patient in the study had different symptoms in both Hebrew and Arabic, indicating that impairment was due to lexical‐level damage, not semantic deficits. Meanwhile, Ibrahim (2011) explores neurocognitive challenges for Arabic‐Hebrew bilinguals. In relevance to Arabic, Ibrahim focuses on how Arabic's diglossia between dialect and MSA can slow reading acquisition and impact academic skills. Also, he identifies how both Arabic and Hebrew need much greater interhemispheric interaction than English because of no vowel markings, unique orthography and concatenative morphology.

Alloush et al. (2024) investigated that early DTI of the left arcuate fasciculus (AF) in aphasic stroke patients can help with language recovery. If the patients' AF was able to be reconstructed, they had better aphasia outcomes than patients without reconstruction. Similarly, Tavano et al. (2009) investigated the long‐term impacts of severe left hemisphere traumatic brain injury (TBI) in a bilingual Arab Italian child. Both studies contribute to understanding the neurolinguistic implications of language recovery.

The review yielded several investigative research studies that focused on Arabic aphasic speakers who were multilingual. For example, Crutch et al. (2006) presented a case report of a bilingual Iraqi with semantic refractory access dysphasia. It investigated the psycholinguistic theory that abstract and concrete concepts used different networks. Similarly, a case study by Jomaa et al. (2022) examined multilingualism's role in post‐stroke aphasia recovery by analysing a trilingual Egyptian woman. Findings showed how code‐switching, cognitive reserve, and multilingualism generally facilitate and further encourage language usage and recovery. Furthermore, Vajramani et al. (2008) looked at bilingual aphasia in the case of a 62‐year‐old bilingual Arabic‐English speaker. The study explored differential language recovery and susceptibility. These studies collectively highlight the complexities of bilingual and multilingual aphasia, emphasizing how linguistic and cognitive factors influence recovery.

In addition, a case study by Taiebine and El Alaoui Faris (2019) was the first documented case of Arabic phonological alexia. It explored documentation in an underreported condition in the Arab world, providing neurolinguistic analysis and contribution to cognitive neuropsychology. Another pioneering research by Taiebine and El Alaoui Faris (2023) investigated the underreported condition Mixed‐Variant Primary Progressive Aphasia (M‐PPA). These studies provide important insights into rare and underexplored language disorders in Arabic‐speaking populations, expanding the field of cognitive neuropsychology in the region.

Also, a few investigative articles contributed to exploring how Arabic aphasia interconnects with syntactic frameworks such as the Tree Pruning Hypothesis (TPH). The concept of this suggests that aphasics struggling with producing and comprehending complex syntactic structures is due to selective impairment at the top of the syntax tree. Friedmann (2002) investigated the topic ‘Question Production in Agrammatism: The Tree Pruning Hypothesis’ to explore if Hebrew‐ and Arabic‐speaking agrammatic aphasics struggled more than English speakers with ‘Wh‐’ questions than ‘Yes/No’ questions. In addition, Friedmann (2001) also analysed agrammatism in Hebrew and Palestinian Arabic‐speaking aphasics to determine if agrammatic deficits can align with syntactic tree structures.

Despite the relative abundance of studies on morphological processing following aphasia, several other linguistic levels also lacked investigation for Arabic aphasia. Syntactic impairment, an important aspect of the Arabic language which also differs considerably from other languages, could benefit from more research being done about it. Another less studied level is semantic processing in Arabic aphasic data. In terms of the methodologies used, the review yielded only 1 neuroimaging study. There were no studies on aphasia accompanying apraxia and dysarthria.

In addition, some papers considered how the sociolinguistic aspects may shape and affect the rehabilitation and diagnosis of aphasics. Alyahya (2024b) had a survey to demonstrate the need for future directions to advocate for aphasia. Earlier, we established the mainstream hold of Western frameworks in aphasic research and tools, and this also is reflected in how educated and informed Arab citizens are regarding speech regression and speech disorders. ’The level of aphasia knowledge in Saudi Arabia was either similar or lower than the reported knowledge in other countries’ was what Alyahya discovered. They identified that only ‘4.79% of those who had heard of aphasia had correct basic knowledge of aphasia related to its features and cause.’ Despite the more socially grounded paper, its presence is necessary, as it underlines the importance of combining research advancement with the advancement of the population's education.

## 5 Discussion

The aim of this review was to assess the body of published research on Arabic‐speaking patients with aphasia, shedding light on methodologies and type of errors investigated in the available literature, and identifying future research trends. Such a review has not been readily available for the Arabic language in recent years. The only other review addressing Arabic data is Beveridge and Bak (2011), in which they carried out a systematic review of articles on aphasia studies in different languages. Articles that were included in their review were published in four leading international journals on aphasia studies only (Aphasiology, Brain and Language, Journal of Neurolinguistics, and Language and Cognitive Processes) between the years 2000 and 2009. They reported that only five studies on Arabic aphasia were published, accounting for only 0.40% of the total literature on aphasia during that period.

By contrast, the current review yielded 48 studies and reviewed data on assessment studies, intervention and therapy studies, and finally investigative studies on linguistic and psycholinguistic theory. The current review provides a bird's eye view of the literature available on Arabic aphasia within the last three decades, focusing on the assessment and intervention studies. Also, there is much added value in our systematic review, because the latest review on Arabic aphasia was done in 2011 (14 years ago), underlining the overwhelming insufficiency of research progression and development as of this moment.

### 5.1 Limitations in Assessment Materials

While this review found that there has been a significant and consistent increase in the development of aphasia assessments for the Arabic language from around 2011 to 2020, there are limitations within the field discussed below. First, an abundance of translations and adaptations of assessments from other languages is still present, as opposed to developing linguistically and culturally driven assessments for the Arabic language. Researchers and clinicians are still heavily depending on assessments used for other languages to create Arabic aphasia assessments. Cultural and linguistic features of the Arabic language do not inform translated and adapted tests.

Second, most of the assessments developed for the Arabic language are screening and short assessments rather than comprehensive. This restricts clinicians from building a comprehensive language profile for their patients, which in turn can affect the intervention designed for each case.

Third, almost all of the existing and newly developed assessments do not control for variables such as imageability, age of acquisition, frequency, familiarity, length, and name agreement when selecting stimuli for their tests, which can be attributed to the lack of normative databases from which test stimuli can be selected for different Arabic varieties. These normative databases are an important part of test development, as stated by Khwaileh and Grosvald (2019), and have only begun to surface in literature for the Arabic language. The significance of matching test stimuli for these factors and other psycholinguistic factors has been supported in research reporting on many languages, including Arabic (e.g., Khwaileh, Body and Herbert 2014; Khwaileh et al. 2018).

This warrants the development of a linguistically and culturally informed aphasia assessment battery for Arabic varieties. A reliable and valid aphasia assessment of language processing needs to be developed from a knowledge base incorporating linguistic and psycholinguistic data from that language. Effective intervention relies on accurate diagnosis through assessment. The need for well‐developed assessment tools is essential for achieving accurate diagnosis, conclusions, and interventions, which will have an impact on patients’ quality of life after stroke.

### 5.2 Scarcity of Intervention Studies

Another outcome of this review is the scarcity of intervention and therapy studies on Arabic aphasia. Only five therapy studies were reported in the literature of Arabic aphasia. The lack of such studies reflects on the services made available to patients with aphasia in the Arab world. This creates a pressing need for therapy and intervention studies for Arabic‐speaking patients, based on linguistic and cultural features that are specific to the Arab world. Unlike assessment studies, which have been growing over the past few years for Arabic aphasia, therapy studies seem to have not seen much, if any, research development or updates on the Arabic language.

The primary challenge is that such an endeavour relies heavily on interdisciplinary work, merging research insights from the fields of psycholinguistics and linguistics with practice‐based expertise. In turn, such research depends on the availability of sufficient time, resources and participants for recruitment, which poses a non‐trivial challenge. A potential solution lies in the incorporation of case‐studies and single subject research designs, as these are less resource‐intensive (Beeson and Robey 2006).

Overarchingly, recognizing the contribution of the fields of psycholinguistics and linguistics in aphasia research is a step in the right direction. It highlights the need to create interdisciplinary research groups, labs and clusters in the Arabic‐speaking world that involve practising clinicians as part of research teams. Without such an arrangement, studies on intervention and therapy would continue to be sparse. A secondary solution for such a challenge is to develop an Arabic aphasia bank for data from Arabic‐speaking patients. This would facilitate access to Arabic aphasia data for researchers worldwide. This is motivated by the small number of researchers across the Arab world and by the fact that patient recruitment is sparse in the Arabic‐speaking world.

### 5.3 An Eye Towards the Different Arabic Varieties

The results also showed that several varieties of Arabic have been investigated over the years, covering those from regions such as the Levant and North Africa. To the best of the authors’ knowledge, there are currently no published investigative studies on aphasia for some of the varieties in other regions, such as Gulf Arabic. Some dialects of Arabic are completely absent from the literature, such as Libyan Arabic. Investigating different varieties might illustrate different error patterns depending on the variety in question (Qorchi and Bouchara 2017 on Moroccan Arabic). This is particularly relevant for the Arabic language due to the fact that different varieties of the language have different morphosyntactic features in many contexts, while the nature of Arabic's diglossia adds more complexity to the situation.

### 5.4 Limitations in Investigative Studies

This review revealed that the majority of studies published on aphasia were investigative in nature. While an in‐depth review of investigative studies is in preparation, limitations in them are of concern here to the extent that they inform clinical practice. There is a dearth of research on the Arabic language in the context of language processing models. Understanding the cognitive and neural underpinnings of comprehension and production breakdown as well as breakdown at the morphological, syntactic and semantic levels underscores the development of valid clinical resources. Current such models are primarily based on English data, and their validity for understanding breakdown in Arabic aphasia remains in question.

From the studies that exist, there is skewness of research towards the production mode and non‐fluent aphasia types. Areas of study that have been well‐developed in other languages but need development for Arabic include comprehension (both at the sentence and single‐word level), syntactic processing (particularly in the context of agrammatism), phonetics and phonology, and lexical retrieval with an eye towards how the unique structural properties of Arabic and its various dialects each level.

## 6 Conclusion

This review confirms the need for further investigations of language breakdown in Arabic aphasia at various linguistic and psycholinguistic levels, the need for linguistically and culturally driven assessments for various Arabic varieties, the urgent need for intervention and therapy studies with Arabic‐speaking patients, and the need to include more aphasia subtypes and aetiologies (including accurate lesion reports) in future research.

Linguistic and psycholinguistic studies have been the predominant approaches used to investigate Arabic aphasia for the last three decades, with their focus being on production rather than comprehension of language. Several levels have lacked investigation for Arabic aphasia, with one of the most needed but understudied levels being syntax and agrammatism.

We further emphasise the fact that current language processing models are primarily derived from English and thus do not account for features of the Arabic language. Developing models to account for cross‐linguistic features would advance our understanding of the nature of aphasia itself. When compared to studies on English and other languages, the current body of Arabic aphasia literature data is fragmented and underrepresented.

Although not yielded in the records of this systematic review, there is at present promising ongoing work to address several of these needs. Digital platforms like SALT Labs house recently developed batteries such as the Arabic Short Aphasia Test (ASAT) and Arabic Comprehensive Aphasia Test (ACAT), which are based on the latest cognitive, neuro‐psycholinguistic and clinical research in Arabic (SALT Labs 2022). In addition, there are ongoing research projects such as ‘Building a Corpus of Arabic Aphasia’ which are aiming to solve the problem of sufficient data availability. This project is compiling a valuable repository of well‐structured, cleaned and annotated Arabic aphasia data for public use to further research in the field (Ulde et al. 2024). These efforts paint a hopeful future for research and development in service of Arabic‐speaking persons with aphasia. They are consistent with a general pattern of growing interest reflected in the consistent and gradual increase in research discourse/literature exchange in the field, indicating that improving both theoretical and practice‐based research is a recognized need. This systematic review is a significant step in the direction of encouraging further development and research for the treatment and diagnosis of Arabic persons with aphasia.

## Conflicts of Interest

The authors declare no conflicts of interest.

## Supporting information



Appendix A: Search String for Systematic Review Methodology & Academic Databases used for Search String

## Data Availability

The data that support the findings of this study are available from the corresponding author upon reasonable request.
