# Supplementary material for: Arabic Aphasia Research Through a Clinical and Linguistic Lens: A Systematic Review of Current Limitations and Future Directions
Source: Int J Lang Commun Disord. 2025 Jun 16;60(4):e70064. doi: 10.1111/1460-6984.70064 (PMC12169208; doi:10.1111/1460-6984.70064)
Supplement: Supplementary file 1 — Appendix A: Search String for Systematic Review Methodology & Academic Databases used for Search String [file JLCD-60-0-s001.docx]

**Appendix:**

**Appendix A: Search String for Systematic Review Methodology & Academic Databases used for Search String**

"Anomic Arabic aphasia" / "Broca’s Arabic aphasia" / "Conduction Arabic aphasia" / "Global Arabic aphasia" / "Mixed transcortical Arabic aphasia" / "Transcortical motor Arabic aphasia" / "Transcortical sensory Arabic aphasia" / "Wernicke’s Arabic aphasia" / "Receptive Arabic aphasia" / "Expressive Arabic aphasia" / "Anomia Arabic aphasia" / "Agrammatism Arabic aphasia" / "Fluent aphasia Arabic aphasia" / "Non-fluent aphasia Arabic aphasia"

| **Databases:** |
| --- |
| PubMed |
| PubPsych |
| APA PsycInfo |
| ScienceDirect |
| Springer Nature Link |
